# Unexpected High Response Rate to Traditional Therapy after Dendritic Cell-Based Vaccine in Advanced Melanoma: Update of Clinical Outcome and Subgroup Analysis

**DOI:** 10.1155/2010/504979

**Published:** 2010-09-27

**Authors:** Laura Ridolfi, Massimiliano Petrini, Laura Fiammenghi, Anna Maria Granato, Valentina Ancarani, Elena Pancisi, Emanuela Scarpi, Massimo Guidoboni, Giuseppe Migliori, Stefano Sanna, Francesca Tauceri, Giorgio Maria Verdecchia, Angela Riccobon, Ruggero Ridolfi

**Affiliations:** ^1^Immunotherapy Unit, Department of Medical Oncology, Istituto Scientifico Romagnolo per lo Studio e la Cura dei Tumori (I.R.S.T.), 47014 Meldola, Italy; ^2^Unit of Biostatistics and Clinical Trials, Department of Medical Oncology, Istituto Scientifico Romagnolo per lo Studio e la Cura dei Tumori (I.R.S.T.), 47014 Meldola, Italy; ^3^Blood Transfusion Unit, Morgagni-Pierantoni Hospital, 47100 Forlì, Italy; ^4^Thoracic Surgery Unit, Morgagni-Pierantoni Hospital, 47100 Forlì, Italy; ^5^Advanced Oncological Surgery Unit, Morgagni-Pierantoni Hospital, 47100 Forlì, Italy

## Abstract

We reviewed the clinical results of a dendritic cell-based phase II clinical vaccine trial in stage IV melanoma and analyzed a patient subgroup treated with standard therapies after stopping vaccination. From 2003 to 2009, 24 metastatic melanoma patients were treated with mature dendritic cells pulsed with autologous tumor lysate and keyhole limpet hemocyanin and low-dose interleukin-2. Overall response (OR) to vaccination was 37.5% with a clinical benefit of 54.1%. All 14 responders showed delayed type hypersensitivity positivity. Median overall survival (OS) was 15 months (95% CI, 8–33). Eleven patients underwent other treatments (3 surgery, 2 biotherapy, 2 radiotherapy, 2 chemotherapy, and 4 biochemotherapy) after stopping vaccination. Of these, 2 patients had a complete response and 5 a partial response, with an OR of 63.6%. Median OS was 34 months (range 16–61). Our results suggest that therapeutic DC vaccination could favor clinical response in patients after more than one line of therapy.

## 1. Introduction

Melanoma accounts for 1%–3% of all malignant tumors and its incidence is increasing in western countries by 6%–7% each year [1–3]. The disease is curable in more than 50% of cases with surgical resection, with an expected 5-year survival of 80%–100% [2]. However, prognosis is poor for patients with advanced disease, with a 5-year life expectancy of <10% and a median survival of 6–8.5 months [4]. Chemotherapy for advanced melanoma continues to be highly unsatisfactory [5, 6]. Dacarbazine (DTIC) is still considered the gold standard of therapy, despite the fact that it has shown “placebo” results in recent phase III trials, obtaining <10% overall response (OR) rates, with an overall survival (OS) of around 6 months [7–9]. Different monochemotherapies and polychemotherapeutic associations, with or without the addition of biological response modifiers, have not proven to be more effective than DTIC [10, 11]. There are no standard second- or third-line therapies. 

The relation between the immune system and the tumor is undoubtedly a complex one. The key ability to distinguish between self and non-self is essential for an adequate response to external pathogens and growing tumor cells. [12] Basic research has identified a number of mechanisms underlying spontaneous antitumor immunity and enabled Dunn to formulate the cancer immunoediting hypothesis, which divides the tumor immune response into three phases: *elimination*, *equilibrium*, and *escape *[13, 14]. 

Both innate and adaptive immunity are involved in the antitumor immune response [15, 16]. In particular, dendritic cells (DCs) play a crucial role in the interplay between innate and adaptive response towards cancer [17]. As members of the innate immune system, their main function is to present antigens to regulate the activation of the adaptive response. DCs can therefore provide signals of both immunostimulation and tolerance to antigen-specific T lymphocytes, thus determining the T response (Th1/Th2) which depends on the activation status of the DCs at the time of the interaction [18, 19]. There is a convincing rationale for the use of DC-based vaccine. In their review of clinical vaccination trials, Rosenberg et al. observed the highest response rate among studies using dendritic cells (7.1%) [20]. Engell-Noerregaard et al. reviewed 38 publications on clinical trials (from 1996 to 2007) using DC-based vaccination in advanced melanoma. The authors reported an objective response rate of 9% with a clinical benefit of 30% (CR+PR+SD) and 21% stable disease. Although a trend was observed between immunological response and overall survival, no definitive conclusions were drawn [21]. 

A combination of immunotherapy with standard treatments (chemotherapy, radiotherapy, and surgery) for cancer is an emerging challenge and an emerging paradigm, in contrast to the concept that defines most standard treatments as immunosuppressive. Examination of combined treatments has yielded unexpected results. Antonia et al. reported a very strong objective response to second-line chemotherapy in nonsmall cell lung cancer patients pretreated with vaccination consisting of dendritic cell (DC) transduced with the full-length wild-type p53. This vaccination was started 8 weeks after completion of first-line chemotherapy [22]. A similar observation was made in patients with follicular B-cell lymphoma vaccinated with an anti-idiotype vaccine while in remission. At disease recurrence, patients were treated with second-line chemotherapy (CHOP schedule) obtaining a much higher partial or complete remission than those expected for this disease [23]. Similarly, Gribben et al. reported unexpected high response rates to salvage therapies after vaccination with universal tumor antigen CYP1B1 in solid tumors in a phase I trial. Five vaccinated patients who developed immunity to the vaccine had a marked objective response to subsequent therapies [24]. In a review of 3 prostate cancer vaccine trials, researchers underlined that vaccinated patients responded better to subsequent chemotherapy than those who had not been vaccinated [25]. The mechanisms responsible for such results remain unknown, although some data have been published on the effect of gemcitabine on myeloid-derived suppressor cells (MDSC) and on the activity of paclitaxel, which binds toll-like receptors (TLRs) to dendritic cells and induces the production of patterns typical of T-helper type I [26].

On the basis of the above, we reviewed and updated the clinical results of a dendritic cell-based clinical vaccine trial in stage IV melanoma patients [27], focusing on a subgroup of 11 patients who underwent other therapies after vaccination.

## 2. Patients and Methods

### 2.1. Patient Population

From June 2003 to December 2007, 24 patients with metastatic melanoma were treated with mature-DCs (mDCs) (10 M/vaccine) pulsed with autologous tumor lysate (ATL) and keyhole limpet hemocyanin (KLH). All but 3 of the patients had been heavily pretreated before vaccination. Patients were vaccinated intradermally with mDCs at the base of the thigh about 10 cm from the groin. Interleukin-2 (IL-2) was administered subcutaneously at a dose of 3 MIU/die on days 3, 4, 5, 6, and 7. The procedure was repeated every 2 weeks for four cycles and monthly thereafter until the lysate was finished or evident progression occurred (symptomatic progression or worsening of clinical conditions with a PS > 2 and absence of signs of immunostimulation). Disease evaluation and immunomonitoring *in vivo* with delayed-type hypersensitivity (DTH) for both ATL and KLH were performed before the first vaccination and every four cycles thereafter. The protocol was approved by the Local Ethics Committee of Forlì Health and Social Services (Azienda USL di Forlì) in 1999.

Prevaccination treatment was as follows: radiotherapy and radical surgery for brain metastases (1 patient), leg perfusion (1), 3 lines of chemotherapy (1), first-line chemotherapy and biochemotherapy after lung metastasis resection (1), high-dose interferon (IFN) for 9 months and bone radiotherapy (1), chemotherapy after low-dose IFN (1) and biochemotherapy (1), or no therapy (2). 

Eleven patients underwent other treatments after stopping vaccination due to disease progression (8 patients) or because the ATL was finished (3, of whom 2 had PR and 1 SD). Of these, 3 underwent high-dose IFN, 2 low-dose IFN, and 6 no treatment. Median disease-free survival (DFS) (from exeresis of primary melanoma to first relapse) was 36 months (range 6–108). Two patients had high LDH levels before vaccination which further increased after treatment. Sites of metastasis after vaccination in the subgroup were lymph nodes (6 patients), soft tissue (7), kidney (2), lung (3), and liver (1). Subsequent treatments were as follows: surgical palliative intervention (3 patients, each undergoing >1 nonradical surgical intervention), biotherapy with anti-CTLA-4 monoclonal antibody in a clinical trial (2 patients, one of whom also received hepatic locoregional fotemustine and chemotherapy with dacarbazine [DTIC] and the other, Gamma Knife radiosurgery for brain metastases and fotemustine), chemotherapy (cisplatin [CDDP] plus DTIC-based polychemotherapy) (2), low-dose biochemotherapy (CDDP+DTIC+IL-2) (2), chemotherapy with CDDP+DTIC and high-dose IL-2 (1), and biochemotherapy (CDDP+DTIC+ low-dose IL-2) plus Gamma Knife radiosurgery (1) (Table 1).

### 2.2. Inclusion Criteria for Vaccine Therapy

Age < 70 years, histologically confirmed diagnosis of melanoma, measurable disease (excluding the presence of brain metastases), previous removal of one or more metastatic lesion from which a sufficient quantity of ATL had been obtained for at least 6 vaccinations, Performance Status (PS) ≤ 2 (according to ECOG criteria), life expectancy > 4 months. Patients who were in good clinical condition (ECOG ≤ 2) but had stopped vaccination due to disease progression or because the ATL was finished were treated with subsequent standard chemotherapy.

### 2.3. Autologous Tumor Lysate (ATL) Preparation

Surgically removed tumor samples were mechanically dispersed to create a single-cell suspension. The largest pieces were incubated at 37°C in enzyme mix (collagenase 0.1%, hyaluronidase 0.01%, DNAse 0.1%, Sigma, Milan, Italy) in RPMI 1640, (PAA Laboratories GmbH, Pasching, Austria) for 3 hours. At the end of incubation the pellets were washed 3 times with phosphate buffered saline (PBS) and incubated for at least 20 minutes in sterile distilled water. Lysis was monitored by light microscope. Larger particles were removed by centrifugation (10 min at 600 g) and the supernatant was passed through a 0.2-*μ*m filter. Protein contents were determined and aliquots were stored at −80°C until use, after verification of sterility.

### 2.4. DC Generation

 DCs were prepared from peripheral blood monocytes (PBMCS) obtained by leukapheresis without previous mobilization. Five to nine liters of blood were processed in each collection. PBMCs were purified on Ficoll-Paque. An aliquot of PBMC was utilized immediately for DC generation and the rest was frozen in bags for use at a later date (4–5 bags/each collection). PBMC were incubated in tissue culture flasks with CellGro DC medium (Cell Genix, Freiburg, Germany) at 10 × 106 cells/mL for 2 hours. The nonadherent cells were discarded and the adherent cells were incubated in CellGro DC medium containing 1000 IU/mL rhIL-4 and 1000 IU/mL rhGM-CSF (Cell Genix, Freiburg, Germany) for 7 days to generate a DC-enriched cell population. On day 6, 90% of the DC culture was pulsed with ATL/ATH (100 mg/mL), while the remaining 10% was pulsed with KLH (50 mg/mL). Both cultures were then incubated overnight. On day 7, the cells were defined as immature DCs (iDCs). After eliminating the previous culture medium, pulsed iDCs were cultured for a further 2 days with a cocktail of cytokines (TNF*α*, IL-1*β*, IL-6, Cell Genix, Freiburg, Germany; PGE2, Pfizer, Italy). On day 9 they were defined as mature DCs (mDC). iDCs or mDcs were removed, washed, and suspended in sterile saline for therapeutic infusion into the patient.

### 2.5. Delayed Type Hypersensitivity

ATL (10 *μ*g) and KLH (5 *μ*g) were each suspended in 500 *μ*L of PBS and injected intradermally into the forearm of the patient. PBS alone was used as negative control. Patients received intradermal injections of ATL, KLH, and physiological solution as negative control at separate sites on the forearm. Eight and twenty-four hours later, DTH was assessed by determining the area of erythema and induration using two-dimensional measurements. The DTH response was considered to be positive if the area of erythema was >10 mm.

### 2.6. Statistical Analysis

Evaluation of response to vaccination was carried out according to modified RECIST criteria, and mixed responses were thus evaluated (decrease in or disappearance of lesions with appearance of new lesions or with modest progression in others). In the event of modest progression, good PS (<2) and positive DTH, vaccination was carried out [28]. Response to postvaccine treatments (11 patients) was carefully evaluated by RECIST criteria, and survival time was calculated as the time between the date of the first vaccination and the date of death from any cause [28] or last followup (June 2009). Overall survival curves were calculated by the Kaplan-Meier method and compared using the Gehan-Wilcoxon test, which tends to weigh the early differences more heavily than other tests belonging to the two-sample rank test family [29].

## 3. Results

### 3.1. Patient Characteristics

Twenty-four patients (13 males and 11 females) with a median age of 50 (range 34–75 years) entered the study. Sites of evaluable metastases were viscera (20 patients), bone (1), soft tissue (14), and lymph nodes (13). Prevaccine treatments were biochemotherapy (12 patients), chemotherapy (7), biotherapy (1) radiotherapy (3), or no therapy (3) (Table 2).

### 3.2. Update of Clinical Vaccine Results

**Figure 1:**
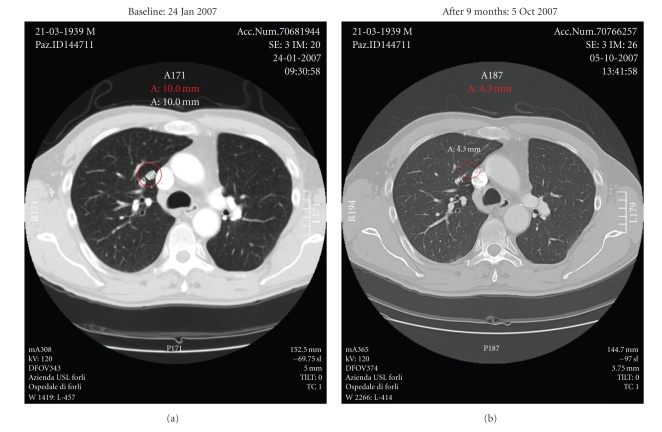
CT scan of patient *n. *25 (BI) before and at the end of vaccination cycles. The red circle indicates the PR observed to vaccine. The patient underwent palliative surgery following progression in adrenal gland and abdominal lymph node metastases after stopping vaccination and has ongoing PR in the lung.

**Figure 2:**
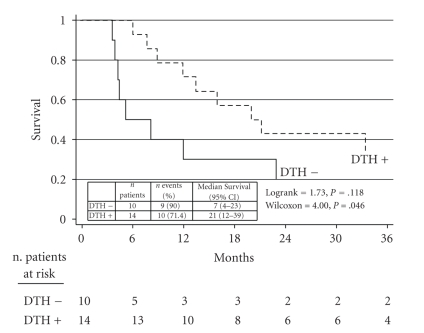
Update of OS curves in 24 vaccinated patients according to DTH (*P* = .046).

**Figure 3:**
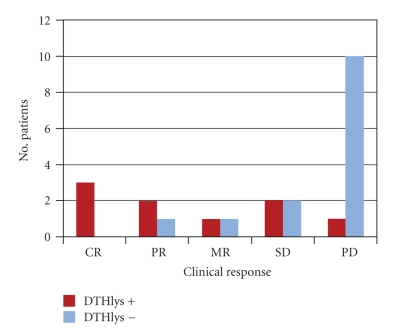
Subdivision of clinical response to DC vaccination on the basis of DTH positivity to lysate (ATL) for the 24 patients treated.

**Figure 4:**
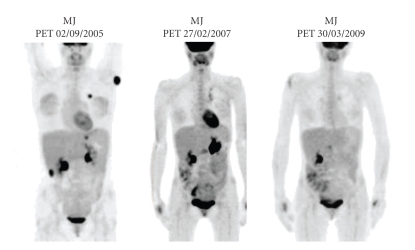
PET/CT scans of patient *n* 19 (MJ) performed in September 2005 (positive PET scan in several metastatic sites), February 2007, and March 2009 (negative PET scan for metasteses). During this period the patient underwent palliative surgery for symptomatic disease (e.g., hematuria due to renal metasteses) or to collect ATL for vaccination that started in February 2005 and terminated in June 2008 because the ATL was finished. Radiosurgery (Gamma Knife) was carried out on a small brain lesion in January 2008. The patient is still in CR (negative PET scan May 2010).

Of the 24 patients treated with vaccine, 2 showed complete response (CR), 2 mixed response (MR), 5 partial response (PR) (Figure 1), and 5 stable disease (SD). The overall response (OR) rate was 37.5% with a clinical benefit of 54.1%. All 14 responders had DTH-positivity to KLH, 10 of whom also to ATL (Table 3). Median overall survival (OS) was 15 months (C.I 95%: 8–33). In a previous study [27], we also observed a statistically significant difference in OS between DTH- positive and DTH-negative patients, which seems to have been maintained, with a median OS of 21 months for DTH+ patients and 7 months for DTH−patients (Gehan-Wilcoxon test; *P* = .046) (Figure 2). Figure 3 shows the subdivision of clinical responses on the basis of DTH positivity to ATL only. Patient 19 obtained a CR (Figure 4) of 15 months (up to June 2009) with alternating non radical surgery and vaccination, as did patients 23 and 25, whose PR is ongoing. These 3 patients are included in the subgroup analysis because surgery was either considered palliative therapy or used to obtain more ATL vaccination or symptom control.

### 3.3. Subgroup (Postvaccine Treatments) Results

**Figure 5:**
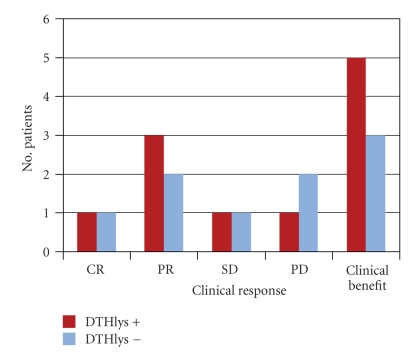
Subdivision of DTH positivity (+) and negativity (−) to lysate (DTH positivity to KLH is contained in the negative group) grouped on the basis of single response and clinical benefit (CR+PR+SD).

The overall response rate to subsequent therapies was 63.6% with 2 CR (1 patient treated with surgery alternating with vaccine and radiosurgery (Gamma Knife) for a small brain metastasis, 1 treated with biochemotherapy plus radiosurgery (Gamma Knife) for a single brain metastasis) and 5 PR (2 patients treated with surgery, 1 patient receiving hepatic locoregional fotemustine+DTIC+anti-CTLA-4 antibody, 1 submitted to DTIC+CDDP and high-dose IL-2, and 1 treated with CDDP+DTIC+ low-dose IL-2), with a median OS of 34 months (median range 16–61). Five of these patients had received one or more treatments before vaccination and experienced an objective response. Of the 11 subgroup patients, 3 had high LDH serum levels after vaccination which normalized during subsequent treatments administered for progressive disease (Table 1). All but one of the patients also had DTH-positivity to at least the KLH test, while 6 were also positive to ATL after vaccination (Figure 5).

### 3.4. Toxicity

 Apart from swelling, redness, and pruritus around the site of inoculation, no noteworthy toxicities or side effects were observed following vaccination. A low fever with mild flu-like symptoms (grade 1-2) was present during administration of IL-2 from days 3 to 7. No autoimmune phenomena were observed apart from the onset of vitiligo in 3 patients, hypothyroidism in 2 and the flaring up of a preexisting vitiligo in one patient (all responders). Toxicity linked to subsequent treatments was coherent with that expected from the different schedules used and no unexpected adverse events were observed.

## 4. Discussion

Systemic therapy for metastatic melanoma remains disappointing and median survival is not improved significantly by currently available chemotherapy regimens [29]. Clinical response rates to most single agents are lower than 15% [30–34], whereas drug combinations have produced response rates of up to 40% [8, 10, 35]. Although a combination of cytotoxic chemotherapy with biological response modifiers such as IL-2 and IFN has resulted in overall response rates of 40%–60% with about 10%–20% CR, biochemotherapy cannot be offered to a substantial proportion of patients with metastatic melanoma because of its high toxicity [11, 36–40]. Furthermore, recent phase III trials have not demonstrated a clear survival benefit from biochemotherapy compared with that obtained with conventional chemotherapy in patients with metastatic melanoma [30, 41–43]. As demonstrated in most of the phase III studies carried out on combination regimens, the incidence of toxicity increases as more drugs are combined and there is no improvement in median survival. New drug regimens are therefore needed with less morbidity than biochemotherapy but more potent antitumor activity than current standard chemotherapies. In fact, understanding how melanoma overcomes host immunity could be the key to developing strategies targeting components of the antitumor immune response, for example, anti-CTLA-4 agents, which has produced encouraging results. Durable objective response rates have been in the range of 4.6%–15% for patients with metastatic melanoma, with a further increment in long-term SD or progression followed by response [44–46].

Specific tumor vaccines attempt to reverse tumor-induced immune suppression and it is thought that they may prolong survival in immune-responsive patients. Vaccines would seem to trigger immunologic memory and thus subsequent treatments that are capable of upregulating tumor-associated antigen expression or of enhancing cross-presentation in a toll-like receptor 4 dependent manner following chemotherapy- or radiotherapy-induced tumor cell death appear to more successful [47]. A progressive surgical reduction of the tumor mass also seems to intensify the effect of the vaccine. 

We observed an OR of 63.6%, which, albeit infrequent in metastatic melanoma after failure of at least one line of therapy, is nevertheless in line with other data published on the treatment of other solid tumors. In our case series, although the 11 patients subjected to postvaccine therapy all had a fairly long initial DFS (median of 36 months) and 5 had also responded to first-line therapy, the response percentage observed after 3-4 lines of therapy was unusually high. It must be underlined that all but one of the 11 patients treated after vaccination had positive DTH to KLH (6/11 also positive to ATL), which seems to support the hypothesis that immunoreactive patients may benefit from being treated even after the failure of vaccine therapy. This highlights the potential usefulness of using vaccine treatment sooner rather than later with the aim of promoting further therapeutic response. In fact, the combination of vaccine with surgery could be effective in reducing the neoplastic mass, facilitating the effect of immunotherapy. Radiotherapy plus vaccination is thought to induce an abscopal effect [48, 49], while chemotherapy, in addition to reducing tumor burden, may induce lymphocytopenia in immunosuppressive cells such as T-regulator lymphocytes [50]. It may even be possible to improve the effect of DC vaccine by combining it with drugs that induce a stronger immunological response (Toll-like receptors) or with agents that inhibit immunosuppression such as antiCTLA-4 antibody. Finally, it would perhaps be useful now to search for predictive factors of immunosuppression, for example, TEM8 expression in dendritic cells. This marker, evaluated at baseline in 4 of the subgroup patients, was low in 3 responders to subsequent therapies and high in one progressive patient, which would seem to confirm previously reported findings by Venanzi et al. [51].

In conclusion, it is clear that the small number of patients and the retrospective setting does not permit any definitive conclusions to be drawn. However, we do feel that our data, together with other findings published in the literature, could form the basis to design new, effective combined treatment strategies. As suggested by other authors [52], it might be useful, for example, to bring forward the time of vaccination with respect to other treatments or to consider the potential of alternating immunotherapy (vaccine alone or vaccine+adjuvant, such as anti-CTLA-4 antibodies) with chemo/radiotherapy or surgery.

## Figures and Tables

**Table 1:** Patients who underwent subsequent therapies after vaccine.

Patient	Adjuvant therapy	DFS (months)	Prior treatment (Prevaccine)	Prevaccine LDH	PostvaccineLDH	Postvaccine treatment	Postvaccine treatment LDH	Best Response	Sites of evaluable metastases
2 P.M.	No	12	BioCT	366	**627**§	CT	**411**	**SD**	Lung, lymph nodes
4 G.D.	HD-IFN	37	no	332	406	CT	310	**PR**	Lymph nodes
						HD-IL-2			
6 T.A.	No	6	CT and RT+surgery for brain metastases	334	281	BioCT	na	**PR**	Soft tissue
8 C.P.	No	48	Locoregional CT (arm)	256	245	BioCT	na	PD	Soft tissue
11 M.J.	LD-IFN	108	CT	193	265	Surgery, Gamma Knife (brain)	384	**C** **R**+*	Lung, kidney, skin, lymph nodes
			Lung surgery, BioCT						
15 B.F	No	57	Arm perfusion, BioCT, surgery	603§	**686§**	Surgery	**433**	**P** **R**+*	Lymph nodes, soft tissue
17 B.I	No	9	BioCT	236	234	Surgery	254	**P** **R**+*	Lung, adrenal gland
18 S.L	HD-INF	36	CT (3 different lines)	374	311	CT, RT (brain), anti-CTLA-4 antibodies	708§	PD	Lymph nodes, soft tissue
22 M.C.	LD-IFN	18	No	591§	**988§**	Hepatic loco-reg CT, DTIC anti-CTLA4ab	**493**	**PR**	Liver, soft tissue
23. R.G	HD-IFN	48	Bone RT	299	313	BioCT,Gamma Knife (brain)	na	**CR+**	Lung, bone
24. B.R	No	24	CT, LD-IFN	236	234	BioCT	na	PD	Lung, soft tissue, lymph nodes

Adj: adjuvant; T: treatment; V: vaccine; HD-IFN: high-dose interferon; BioCT: biochemotherapy; CT: chemotherapy; RT: radiotherapy; LD: low dose; anti-CTLA4ab, anti-CTLA4 monoclonal antibody; §, elevated; *, responses obtained alternating palliative surgery with vaccine.

**Table 2:** Patient characteristics.

Median age 50 years (range 34–75)	Patients (*n* = 24) *n* * (%) *
*Sex*	
Male	13 (54.2)
Female	11 (45.8)

*Sites of evaluable disease*	
Viscera	20
Bone	1
Soft tissue	14
Lymph nodes	13

*Previous treatments*	
BioT	1
BioCT	12
CT	7
RT	3
No treatment	3

**Table 3:** Update (June 2009) of results on 24 patients treated with mDC vaccine.

Patient ID	*n* vaccinations	DTH Best response after 4 or more vaccinations		Vitiligo	Clinical response	Response duration	OS (Months)
		ATL	KLH				
(1) P.M	7	−	−		PR	3	14
(2) P.M	15	++	++++		MR	6	22
(3) R.L.	10	−	++		SD	9	14
(4) G.D.	16	++	+++	+	CR	8	34
(5) R.G.	4	−	+++		PD	—	8
(6) T.A.	13	−	++		MR	12	41
(7) B.A.	4	−	−		PD	—	7
(8) C.P.	6	−	++		PD	—	20
(9) O.M.	4	−	−		PD	—	5
(10) LB.	4	−	−		PD	—	3
(11) M.J.	8+8+8+4	+	+++	+	CR	15+*	52+
(12) O.G.	5	−	−		PD	—	3
(13) M.R.	4	+	++		PD	—	6
(14) DiI.G	10	+	+		CR	36+	39+
(15) B.F	21	++	++		PR	20+*	40+
(16) I.I	6	−	−		PD	—	7
(17) B.I	4	++	−/+	+	PR	21+*	31+
(18) S.L	9	−	+		SD	7	22
(19) N.F.	6	++	+		PR	5	8
(20) B.R	12	+++	+++		SD	8	10
(21) S.M	11	−	−		PD	—	12
(22) M.C	6	−	+		PD	—	61+
(23) R.G	5	−	−		PD	—	60+
(24) B.R	13	+	+++		SD	11	16

PR: partial response; MR: mixed response; SD: stable disease; CR: complete response; PD: progressive disease; OS: overall survival; DTH: delayed-type hypersensitivity test; *responses obtained with palliative surgery + vaccine alternating; Grey area, 11 patients given postvaccine therapy.

## References

[1] Baade P, Coory M (2005). Trends in melanoma mortality in Australia: 1950–2002 and their implications for melanoma control. *Australian and New Zealand Journal of Public Health*.

[2] Parkin DM, Bray F, Ferlay J, Pisani P (2005). Global cancer statistics, 2002. *Ca-A Cancer Journal for Clinicians*.

[3] Tsao H, Atkins M, Sober A (2004). Management of cutaneous melanoma. *New England Journal of Medicine*.

[4] Crosby T, Fish R, Coles B, Mason MD (2000). Systemic treatments for metastatic cutaneous melanoma. *Cochrane Database of Systematic Reviews*.

[5] Lee ML, Tomsu K, Von Eschen KB (2000). Duration of survival for disseminated malignant melanoma: results of a meta-analysis. *Melanoma Research*.

[6] Bajetta E, Del Vecchio M, Bernard-Marty C (2002). Metastatic melanoma: chemotherapy. *Seminars in Oncology*.

[7] Flaherty KT (2006). Chemotherapy and targeted therapy combinations in advanced melanoma. *Clinical Cancer Research*.

[8] Chapman PB, Einhorn LH, Meyers ML (1999). Phase III multicenter randomized trial of the Dartmouth regimen versus dacarbazine in patients with metastatic melanoma. *Journal of Clinical Oncology*.

[9] Schadendorf D, Ugurel S, Schuler-Thurner B (2006). Dacarbazine (DTIC) versus vaccination with autologous peptide-pulsed dendritic cells (DC) in first-line treatment of patients with metastatic melanoma: a randomized phase III trial of the DC study group of the DeCOG. *Annals of Oncology*.

[10] Huncharek M, Caubet JF, McGarry R (2001). Single-agent DTIC versus combination chemotherapy with or without immunotherapy in metastatic melanoma: a meta-analysis of 3273 patients from 20 randomized trials. *Melanoma Research*.

[11] Keilholz U, Gore ME (2002). Biochemotherapy for advanced melanoma. *Seminars in Oncology*.

[12] Pardoll D (2003). Does the immune system see tumors as foreign or self?. *Annual Review of Immunology*.

[13] Dunn GP, Old LJ, Schreiber RD (2004). The immunobiology of cancer immunosurveillance and immunoediting. *Immunity*.

[14] Swann JB, Smyth MJ (2007). Immune surveillance of tumors. *Journal of Clinical Investigation*.

[15] Ferrone S, Whiteside TL (2007). Tumor Microenvironment and Immune Escape. *Surgical Oncology Clinics of North America*.

[16] Whiteside TL (2008). The tumor microenvironment and its role in promoting tumor growth. *Oncogene*.

[17] Banchereau J, Steinman RM (1998). Dendritic cells and the control of immunity. *Nature*.

[18] Lanzavecchia A, Sallusto F (2001). Regulation of T cell immunity by dendritic cells. *Cell*.

[19] Shurin MR (1996). Dendritic cells presenting tumor antigen. *Cancer Immunology Immunotherapy*.

[20] Rosenberg SA, Yang JC, Restifo NP (2004). Cancer immunotherapy: moving beyond current vaccines. *Nature Medicine*.

[21] Engell-Noerregaard L, Hansen TH, Andersen MH, Thor Straten P, Svane IM (2009). Review of clinical studies on dendritic cell-based vaccination of patients with malignant melanoma: assessment of correlation between clinical response and vaccine parameters. *Cancer Immunology, Immunotherapy*.

[22] Antonia SJ, Mirza N, Fricke I (2006). Combination of p53 cancer vaccine with chemotherapy in patients with extensive stage small cell lung cancer. *Clinical Cancer Research*.

[23] Inogès S, Rodrìguez-Calvillo M, Zabalegui N (2006). Clinical benefit associated with idiotypic vaccination in patients with follicular lymphoma. *Journal of the National Cancer Institute*.

[24] Gribben JG, Ryan DP, Boyajian R (2005). Unexpected association between induction of immunity to the universal tumor antigen CYP1B1 and response to next therapy. *Clinical Cancer Research*.

[25] Schlom J, Arlen PM, Gulley JL (2007). Cancer vaccines: moving beyond current paradigms. *Clinical Cancer Research*.

[26] Ramakrishnan R, Antonia S, Gabrilovich DI (2008). Combined modality immunotherapy and chemotherapy: a new perspective. *Cancer Immunology, Immunotherapy*.

[27] Ridolfi R, Petrini M, Fiammenghi L (2006). Improved overall survival in dendritic cell vaccination-induced immunoreactive subgroup of advanced melanoma patients. *Journal of Translational Medicine*.

[28] Hoos A, Parmiani G, Hege K (2007). A clinical development paradigm for cancer vaccines and related biologics. *Journal of Immunotherapy*.

[29] Marubini E, Valsecchi MG, Barnett V (1995). Analysing survival data from clinical trials and observational studies. *Statistics in Practice*.

[30] Middleton MR, Grob JJ, Aaronson N (2000). Randomized phase III study of temozolomide versus dacarbazine in the treatment of patients with advanced metastatic malignant melanoma. *Journal of Clinical Oncology*.

[31] Atkins MB (1997). The treatment of metastatic melanoma with chemotherapy and biologics. *Current Opinion in Oncology*.

[32] Legha SS, Ring S, Papadopoulos N, Raber M, Benjamin RS (1990). A phase II trial of taxol in metastatic melanoma. *Cancer*.

[33] Bedikian AY, Weiss GR, Legha SS (1995). Phase II trial of docetaxel in patients with advanced cutaneous malignant melanoma previously untreated with chemotherapy. *Journal of Clinical Oncology*.

[34] Avril MF, Aamdal S, Grob JJ (2004). Fotemustine compared with dacarbazine in patients with disseminated malignant melanoma: a phase III study. *Journal of Clinical Oncology*.

[35] Buzaid AC (1993). Cisplatin, vinblastine, and dacarbazine versus dacarbazine alone in metastatic melanoma: preliminary results of a phase III Cancer Community Oncology Program (CCOP) trial. *Proceedings of the American Society of Clinical Oncology*.

[36] Atkins MB, O’Boyle KR, Sosman JA (1994). Multiinstitutional phase II trial of intensive combination chemoimmunotherapy for metastatic melanoma. *Journal of Clinical Oncology*.

[37] Atzpodien J, Lopez Hänninen E, Kirchner H (1995). Chemoimmunotherapy of advanced malignant melanoma: sequential administration of subcutaneous interleukin-2 and interferon-*α* after intravenous dacarbazine and carboplatin or intravenous dacarbazine, cisplatin, carmustine and tamoxifen. *European Journal of Cancer A*.

[38] Gibbs P, Iannucci A, Becker M (2000). A phase II study of biochemotherapy for the treatment of metastatic malignant melanoma. *Melanoma Research*.

[39] Legha SS, Ring S, Bedikian A (1996). Treatment of metastatic melanoma with combined chemotherapy containing cisplatin, vinblastine and dacarbazine (CVD) and biotherapy using interleukin-2 and interferon-. *Annals of Oncology*.

[40] Chiarion-Sileni V, Del Bianco P, de Salvo GL (2003). Quality of life evaluation in a randomised trial of chemotherapy versus bio-chemotherapy in advanced melanoma patients. *European Journal of Cancer*.

[41] Rosenberg SA, Yang JC, Schwartzentruber DJ (1999). Prospective randomized trial of the treatment of patients with metastatic melanoma using chemotherapy with cisplatin, dacarbazine, and tamoxifen alone or in combination with interleukin-2 and interferon alfa-2b. *Journal of Clinical Oncology*.

[42] Ives NJ, Stowe RL, Lorigan P, Wheatley K (2007). Chemotherapy compared with biochemotherapy for the treatment of metastatic melanoma: a meta-analysis of 18 trials involving 2,621 patients. *Journal of Clinical Oncology*.

[43] Ridolfi R, Chiarion-Sileni V, Guida M (2002). Cisplatin, dacarbazine with or without subcutaneous interleukin-2, and interferon alfa-2b in advanced melanoma outpatients: results from an Italian multicenter phase III randomized clinical trial. *Journal of Clinical Oncology*.

[44] Weber JS (2006). The clinical utility of cytotoxic T lymphocyte antigen 4 abrogation by human antibodies. *Melanoma Research*.

[45] Ridolfi L, Ridolfi R (2009). Anti-CTLA-4 therapy in melanoma: role of ipilimumab (MDX-010). *Expert Review of Dermatology*.

[46] O’Day SJ, Hamid O, Urba WJ (2007). Targeting cytotoxic T-lymphocyte antigen-4 (CTLA-4): a novel strategy for the treatment of melanoma and other malignancies. *Cancer*.

[47] Apetoh L, Ghiringhelli F, Tesniere A (2007). Toll-like receptor 4-dependent contribution of the immune system to anticancer chemotherapy and radiotherapy. *Nature Medicine*.

[48] Demaria S, Ng B, Devitt ML (2004). Ionizing radiation inhibition of distant untreated tumors (abscopal effect) is immune mediated. *International Journal of Radiation Oncology Biology Physics*.

[49] Demaria S, Bhardwaj N, McBride WH, Formenti SC (2005). Combining radiotherapy and immunotherapy: a revived partnership. *International Journal of Radiation Oncology Biology Physics*.

[50] Ghiringhelli F, Larmonier N, Schmitt E (2004). CD4+CD25+ regulatory T cells suppress tumor immunity but are sensitive to cyclophosphamide which allows immunotherapy of established tumors to be curative. *European Journal of Immunology*.

[51] Venanzi FM, Petrini M, Fiammenghi L (2010). Tumor endothelial marker 8 expression levels in dendritic cell-based cancer vaccines are related to clinical outcome. *Cancer Immunology, Immunotherapy*.

[52] Zitvogel L, Apetoh L, Ghiringhelli F, Kroemer G (2008). Immunological aspects of cancer chemotherapy. *Nature Reviews Immunology*.

