# Erratum to “Unexpected High Response Rate to Traditional Therapy after Dendritic Cell-Based Vaccine in Advanced Melanoma: Update of Clinical Outcome and Subgroup Analysis”

**DOI:** 10.1155/2011/283896

**Published:** 2011-05-03

**Authors:** Laura Ridolfi, Massimiliano Petrini, Laura Fiammenghi, Anna Maria Granato, Valentina Ancarani, Elena Pancisi, Emanuela Scarpi, Massimo Guidoboni, Giuseppe Migliori, Stefano Sanna, Francesca Tauceri, Giorgio Maria Verdecchia, Angela Riccobon, Linda Valmorri, Ruggero Ridolfi

**Affiliations:** ^1^Immunotherapy Unit, Department of Medical Oncology, Scientifc Institute of Romagna for the Study and Treatment of Cancer (I.R.S.T.), 47014 Meldola, Italy; ^2^Unit of Biostatistics and Clinical Trials, Scientifc Institute of Romagna for the Study and Treatment of Cancer (I.R.S.T.), 47014 Meldola, Italy; ^3^Blood Transfusion Unit, Morgagni-Pierantoni Hospital, 47014 Forlì, Italy; ^4^Thoracic Surgery Unit, Morgagni-Pierantoni Hospital, 47014 Forlì, Italy; ^5^Advanced Oncological Surgery Unit, Morgagni-Pierantoni Hospital, 47014 Forlì, Italy

The authors would like to add Dr. Linda Valmorriat to the authors' list. Thus, the authors' list will be: Laura Ridolfi, Massimiliano Petrini, Laura Fiammenghi, AnnaMaria Granato, Valentina Ancarani, Elena Pancisi, Emanuela Scarpi, Massimo Guidoboni, Giuseppe Migliori, Stefano Sanna, Francesca Tauceri, Giorgio Maria Verdecchia, Angela Riccobon, Linda Valmorriat, and Ruggero Ridolfi.

